# Antimicrobial Resistance in Companion Animals: A New Challenge for the One Health Approach in the European Union

**DOI:** 10.3390/vetsci9050208

**Published:** 2022-04-24

**Authors:** Ana Marco-Fuertes, Clara Marin, Laura Lorenzo-Rebenaque, Santiago Vega, Laura Montoro-Dasi

**Affiliations:** Departamento de Producción y Sanidad Animal, Salud Pública Veterinaria y Ciencia y Tecnología de los Alimentos, Instituto de Ciencias Biomédicas, Facultad de Veterinaria, Universidad Cardenal Herrera-CEU, CEU *Universities*, Avenida Seminario s/n, 46113 Moncada, Spain; ana.marco3@alumnos.uchceu.es (A.M.-F.); clara.marin@uchceu.es (C.M.); laura.lorenzorebenaque@uchceu.es (L.L.-R.); laura.montoro@uchceu.es (L.M.-D.)

**Keywords:** antimicrobial resistance, companion animals, One Health, public health, surveillance programmes, monitoring programmes

## Abstract

Antimicrobial resistance (AMR) and the increase in multi-resistant bacteria are among the most important threats to public health worldwide, according to the World Health Organisation (WHO). Moreover, this issue is underpinned by the One Health perspective, due to the ability of AMR to be transmitted between animals and humans living in the same environment. Therefore, since 2014 different surveillance and control programmes have been established to control AMR in commensal and zoonotic bacteria in production animals. However, public health authorities’ reports on AMR leave out companion animals, due to the lack of national programmes and data collection by countries. This missing information constitutes a serious public health concern due to the close contact between companion animals, humans and their surrounding environment. This absence of control and harmonisation between programmes in European countries leads to the ineffectiveness of antibiotics against common diseases. Thus, there is a pressing need to establish adequate surveillance and monitoring programmes for AMR in companion animals and further develop alternatives to antibiotic use in this sector, considering the impact this could have on the gut microbiota. In this context, the aim of this review is to evaluate the current control and epidemiological situations of AMR in companion animals in the European Union (EU), as well as the proposed alternatives to antibiotics.

## 1. Introduction

Antimicrobial resistance (AMR) is currently one of the main concerns worldwide, signalled by the World Health Organisation (WHO) as one of the top 10 global public health threats in 2019 [1]. Indeed, the prevalence of multi-resistant bacteria and the difficulty of treating bacterial infections in both animals and humans have increased in recent years [1,2]. Moreover, AMR is considered a One Health issue, as it englobes animal, human and environmental health [1,3]. In this context, companion animals are particularly relevant due to their growing population, with more than 60 million cats and dogs in the European Union (EU), and their close contact with people, animals and their surrounding environment [4].

Due to the importance of AMR, governments worldwide have been involved in the search for solutions and have established surveillance programmes to monitor AMR prevalence and evolution in zoonotic and commensal bacteria, considered potential reservoirs of resistant genes [5]. Finally, in 2020 the Global Leaders Group (GLG) on AMR was formed, with the main objective of controlling AMR bacteria in different sectors covering human, animal and environmental health [6].

In the EU, the European Food Safety Authority (EFSA) coordinates surveillance programmes for commensal bacteria in food-producing animals, along with the monitoring of zoonotic agents and their AMR [5]. In 2009, the European Medicines Agency (EMA) rolled out the European Surveillance of Veterinary Antimicrobial Consumption (ESVAC) network to gather information on antibiotic consumption (AMC) in animals across the EU [7]. Subsequently, the European Centre for Disease Prevention and Control (ECDC) and the EFSA joined forces with the EMA to establish the Joint Inter-Agency Analysis of Antimicrobial Consumption and Antimicrobial Resistance (JIACRA), which reports findings on AMC and AMR in humans and food-producing animals [7]. These programmes have achieved significant reductions in the use of antibiotics in food-producing animals, without negative effects on production or profits [8,9].

However, the focus has always been on food-producing animals because of their importance as transmitters of AMR through the food chain. These programmes have achieved a significant reduction in antibiotic consumption, so the next step is to focus all efforts on companion animals [1]. Currently, only a few countries have specific control programmes for AMR and AMC in this sector [10]. As reported above, the missing information on the overall AMR situation could pose serious problems for public health due to the close contact between owners and companion animals [11]. Furthermore, in veterinary clinics and hospitals, antibiotics reserved for human medicine are permitted [9]. The scarce information available on AMR in companion animals only includes dogs and cats, and other companion animal species such as birds or rabbits are left out. The information gathered on these animals comes from some studies conducted by different research groups [12,13,14,15].

In this context, the EU plans to launch the European Antimicrobial Resistance Surveillance Network in Veterinary Medicine (EARS-Vet) to get an overview of the current situation and establish European AMR monitoring systems [5]. Therefore, this review assesses the current epidemiological situation of AMR in companion animals in the EU and the alternatives to antibiotic use developed in clinical veterinary practice.

## 2. Methodology

An exhaustive literature search was conducted in which different sources of information were reviewed and evaluated to address the current status of AMR surveillance programmes in companion animals. 

The search was performed through search engines such as PubMed, Google Scholar and Scopus. In addition, reports and publications of health organisations such as the WHO, OIE (World Organisation for Animal Health), EFSA, ECDC or EMA were reviewed. 

This review included articles written and published in English and German, as the annual AMRs report submitted by Germany was written in their native language. The search terms that provided most of the information were: “Antimicrobial resistant”, “Companion animals”, “AMR surveillance programmes in Europe”, “AMR monitoring programmes in Europe”, “EARS-Vet”, “One Health”. Moreover, all articles were filtered to be no older than 2018, except for one article about a specific clinical case from 2009. 

## 3. AMR Surveillance and Monitoring Programmes

### 3.1. Global AMR Situation

The current imbalance between AMR programmes and reported data limit the full understanding of an integrated One Health global AMR surveillance system [16]. In 2015, to address this issue, the Joint Tripartite formed by the Food and Agriculture Organisation of the United Nations, the World Organisation for Animal Health and the WHO adopted a Global Action Plan to ensure worldwide capacity to control and prevent AMR bacterial infectious diseases, with effective and safe medicines used responsibly and accessible to the world population [17]. Since then, the Joint Tripartite has been working to establish an integrated surveillance system platform to collect and assemble data reported by countries related to human, animal, food and environmental health. They also assess the implementation and development of the Global Action Plan on AMR in all sectors [17].

In the global scope, there are different programmes to control AMR in both production and companion animals. On the one hand, there are AMR Surveillance Programmes that study bacterial species present in animal infections and their AMR [18,19]. On the other hand, there are AMR Monitoring Programmes that take samples from healthy animals to study commensal bacteria as reservoirs of resistance genes [17,18,19]. However, in order to control AMR under the One Health perspective, it is also mandatory to start monitoring AMR in companion animals and to monitor AMC [7].

### 3.2. AMR Surveillance Programmes in Europe

In the EU, the EFSA coordinates the AMR Surveillance Programmes in food-producing animals, in accordance with Directive 2003/99/EC and Commission Implementing Decision (EU) 2020/1729 [5]. Moreover, as of 2019 the EMA analyses the sales and use of antimicrobial products in animals, following the guidelines set out in Regulation (EU) 2019/6, which updates and repeals the previous legislation Directive 2001/82/EC [5]. 

Finally, the JIACRA brings together all available data on AMR and AMC, comparing animal and human results. In the last report, AMC was lower in food-producing animals than in humans, with 108 mg/Kg and 130 mg/Kg of estimated biomass, respectively. These results show the effectiveness of the current surveillance and monitoring programmes in place in the animal production sector [7]. 

Moreover, the EMA categorised antimicrobials according to the risk they pose to public health and the need for their use in veterinary medicine. These are divided into four categories: Category D, which should be used with caution and as a first line of treatment whenever possible; Category C, with antimicrobials that should be used with caution and only when Category D antibiotics fail clinically; Category B, including antimicrobials that are critically important in human medicine and whose use should be restricted in veterinary medicine and adopted only when all therapeutic alternatives (D and C) have been exhausted; and finally, Category A, whose use is limited in human medicine and is not authorised in the European Union (EU) in veterinary medicine and therefore for the treatment of production animals [20]. However, there are some exceptions in the clinical care of companion animals, because in exceptional situations, antimicrobials from category A could be dispensed and used, hence creating a serious problem [20].

Antimicrobial use (AMU) in companion animals is also not included in the annual reports conducted by the EMA (at a European level) [20] and by the OIE (at a global level) [21] due to the lack of available data on the cat and dog populations, but a few countries included the sale of antimicrobials for companion animals as part of their surveillance systems. The remainder of the data in the reports were from food-producing animals.

Moreover, only a few countries submit reports that include AMR in companion animals that live in close contact with humans and are considered an important potential source of AMR. These established AMR Surveillance Programmes are mainly focused on AMR prevalence in pathogenic bacteria [10]. In this sense, *Staphylococcus pseudintermedius* as well as *Staphylococcus aureus* are coagulase-positive species of *Staphylococcus*. These bacterial species are opportunistic pathogens found in the mucous membranes of animals as part of the commensal bacteria and have been reported to infect humans [22]. On the other hand, *Escherichia coli* is found in the normal flora of the gastrointestinal tract in both animals and humans [23]. However, it is one of the most common aetiologies in digestive pathologies and is the most frequently isolated bacterium in kidney and urogenital tract infections (UTI) in dogs and cats [24]. Thus, *E. coli* could serve as an important source of infections and resistance genes for humans [24,25]. However, while bacterial species can infect animals and humans in both directions, another consideration to bear in mind is that antibiotic resistance genes are spread by animals and humans through their shared environment [23,25]. 

**Figure 1:**
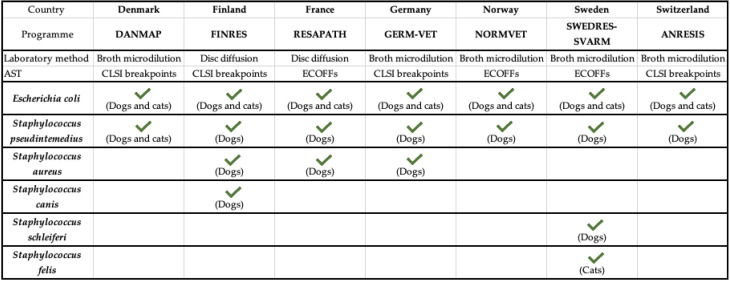
Summary of AMR surveillance programmes in the European Union. AST: ANTIBIOTIC Susceptibility Test; CLSI: Clinical Laboratory Standards Institute. Adapted from [10]. √: it indicates the bacteria included in each AMR surveillance program.

In the following subsections, AMR surveillance programmes and AMU data for the different EU countries are described. All the information regarding animal sampling, bacterial species analysed and laboratory methodology is summarised in Figure 1.

#### 3.2.1. Denmark (DANMAP)

DANMAP is the Danish programme for monitoring AMC and AMR in bacteria from humans, food-producing animals and food [26]. There is no specific AMR monitoring system for companion animals, but the latest report included data from a study carried out in the country between 2012 and 2019 on isolates obtained from samples submitted to the Veterinary Diagnostic Microbiology Laboratory at the University of Copenhagen [10]. 

In Denmark, AMU is estimated as kg of active compound sold for dogs and cats. However, it is true that the data for companion animals are not as precise as those for food-producing animals, as no distinction is made by species [26]. In total, 1232 kg of active substances were sold in 2020, of which more than 680 kg were compounds of the penicillin group, and 271 kg of the sulphonamides–trimethoprim group. While it is true that the use of cephalosporins has decreased by 68% since 2012, 100% of the dispensed third- and fourth-generation cephalosporins were used in dogs and cats [26]. 

**Figure 2:**
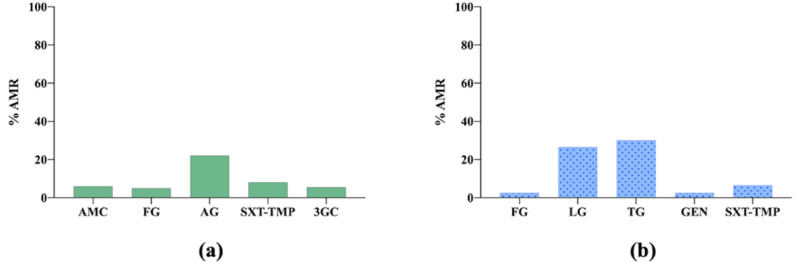
(**a**) Antimicrobial resistance (AMR) prevalence in *Escherichia coli* isolated from dogs and cats in Denmark. AMC: amoxicillin–clavulanic acid; FG: fluoroquinolones group; AG: aminopenicillins group, SXT-TMP: sulfamethoxazole–trimethoprim; 3GC: third-generation cephalosporins. (**b**) Antimicrobial resistance (AMR) prevalence in *Staphylococcus pseudintermedius* isolated from dogs and cats in Denmark. FG: fluoroquinolones group; LG: lincosamides group; TG: tetracyclines group; GEN: gentamicin; SXT-TMP: sulfamethoxazole–trimethoprim.

In this study, no major overall changes were found over time, and the results of AMR in cats and dogs are shown together as one (Figure 2) [10].

#### 3.2.2. Finland (FINRES)

FINRES stands for Finnish Veterinary Antimicrobial Resistance Monitoring and Consumption of Antimicrobial Agents [27]. The samples included in this study were submitted to the Veterinary Medicine Faculty at the University of Helsinki from both the Veterinary Hospital of the University and private veterinary clinics (approximately 60%) [10]. 

AMU data for companion animals in Finland are not available. The report only includes information on food-producing animals [27].

**Figure 3:**
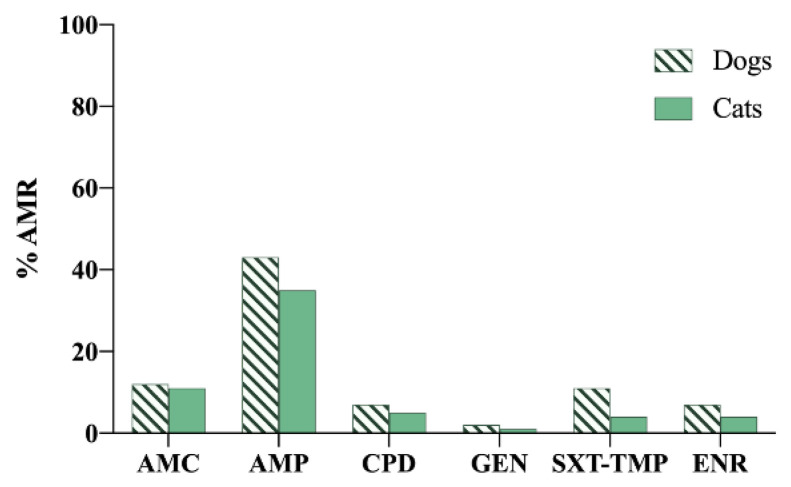
Antimicrobial resistance (AMR) prevalence in *Escherichia coli* isolated from dogs and cats in Finland. AMC: amoxicillin–clavulanic acid; AMP: ampicillin; CPD: cefpodoxime; GEN: gentamicin; SXT-TMP: sulfamethoxazole–trimethoprim; ENR: enrofloxacin.

**Figure 4:**
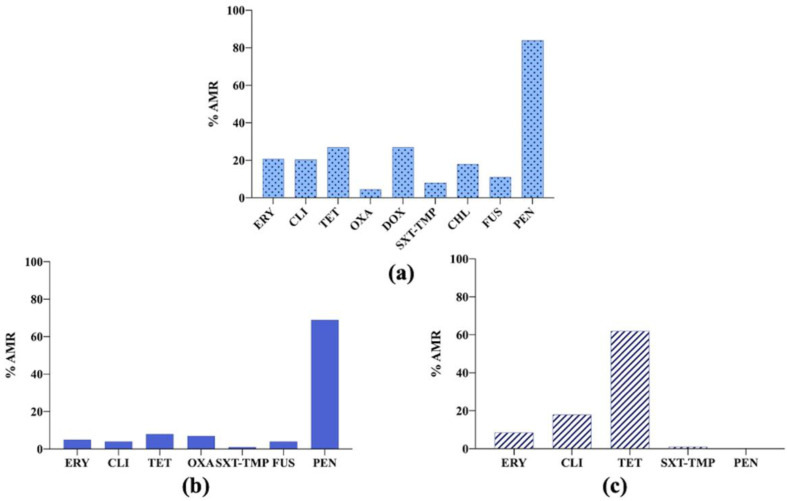
(**a**) Antimicrobial resistance (AMR) prevalence in *Staphylococcus pseudintermedius* isolated from dogs in Finland. ERY: erythromycin; CLI: clindamycin; TET: tetracycline; OXA: oxacillin; DOX: doxycycline; SXT-TMP: sulfamethoxazole–trimethoprim; CHL: chloramphenicol; FUS: fusidic acid; PEN: penicillin. (**b**) Antimicrobial resistance (AMR) prevalence in *Staphylococcus aureus* isolated from dogs in Finland. ERY: erythromycin; CLI: clindamycin; TET: tetracycline; OXA: oxacillin; SXT-TMP: sulfamethoxazole–trimethoprim; FUS: fusidic acid; PEN: penicillin. (**c**) Antimicrobial resistance (AMR) prevalence in *Staphylococcus canis* isolated from dogs in Finland. ERY: erythromycin; CLI: clindamycin; TET: tetracycline; SXT-TMP: sulfamethoxazole–trimethoprim; PEN: penicillin.

Regarding the main results obtained in 2020, it is important to highlight the presence of oxacillin, resistance to which was found in *S. aureus* and *S. pseudintermedius* isolates, which indicated the presence of methicillin-resistant *Staphylococcus aureus* (MRSA) and methicillin-resistant *Staphylococcus pseudintermedius* (MRSP), respectively [22,28], as oxacillin has replaced methicillin in clinical use because they have a similar composition. However, for *Staphylococcus canis*, all the isolates analysed were sensitive to penicillin [27] (Figure 3 and Figure 4). 

#### 3.2.3. France (RESAPATH)

RESAPATH is the French surveillance network for AMR in bacteria obtained from diseased animals. The network is coordinated by the French Agency for Food, Environmental and Occupational Health and Safety and collects data from 71 laboratories registered in this programme [29].

In France, 20.4 tonnes of antimicrobials were sold for companion animals in 2020. Animal exposure to antimicrobials has been gradually decreasing, except for the penicillin group, whose use has increased in recent years, reaching values similar to those of 2011 [30].

**Figure 5:**
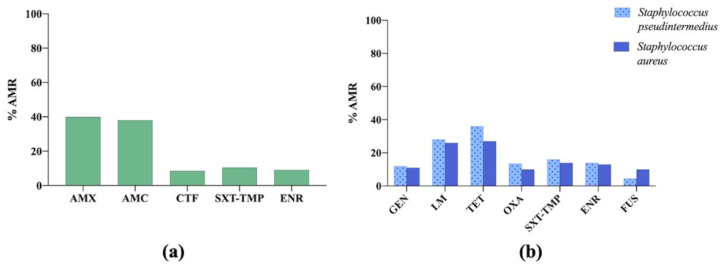
(**a**) Antimicrobial resistance (AMR) prevalence in *Escherichia coli* isolated from dogs and cats in France. AMX: amoxicillin; AMC: amoxicillin–clavulanic acid; CTF: ceftiofur; SXT-TMP: sulfamethoxazole–trimethoprim; ENR: enrofloxacin. (**b**) Antimicrobial resistance (AMR) prevalence in *Staphylococcus pseudintermedius* and *Staphylococcus aureus* isolated from dogs in France. GEN: gentamicin; LM: lincomycin; TET: tetracycline; OXA: oxacillin; SXT-TMP: sulfamethoxazole–trimethoprim; ENR: enrofloxacin; FUS: fusidic acid.

The results of the latest report showed a high AMR level to amoxicillin and amoxicillin–clavulanic acid in *E. coli* isolates. Moreover, the percentage of resistant *S. pseudintermedius* isolates was high for all the antibiotics tested [29] (Figure 5).

#### 3.2.4. Germany (GERM-Vet)

GERM-Vet reports the AMR prevalence in clinically relevant animal pathogenic bacteria according to the Federal Office of Consumer Protection and Food Safety (BVL), which includes public, private and university laboratories [10,31]. 

In the latest report, AMU showed a continuous decrease to 670 tonnes of total antimicrobials sold. It is important to highlight that the sales of compounds of the fluoroquinolones group and of third- and fourth- generation cephalosporins reached the lowest value since 2011, but all these active substances cannot be linked to any specific species, as the use of some antimicrobials is approved for many animal species [32]. 

**Figure 6:**
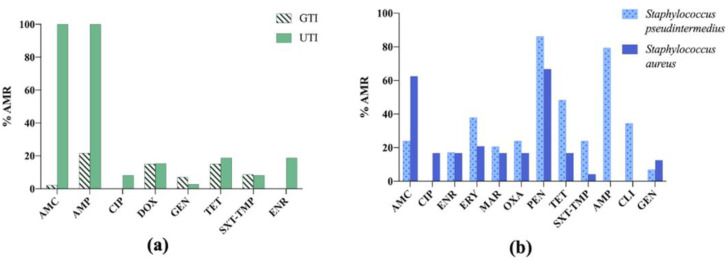
(**a**) Antimicrobial resistance (AMR) prevalence in *Escherichia coli* isolated from dogs and cats in Germany. GTI: gastrointestinal tract infections; UTI: urinary tract infections; AMC: amoxicillin–clavulanic acid; AMP: ampicillin; CIP: ciprofloxacin; DOX: doxycycline; GEN: gentamicin; TET: tetracycline; SXT-TMP: sulfamethoxazole–trimethoprim; ENR: enrofloxacin. (**b**) Antimicrobial resistance (AMR) prevalence in *Staphylococcus pseudintermedius* and *Staphylococcus aureus* isolated from dogs in Germany. AMC: amoxicillin –clavulanic acid; CIP: ciprofloxacin; ENR: enrofloxacin; ERY: erythromycin; MAR: marbofloxacin; OXA: oxacillin; PEN: penicillin; TET: tetracycline; SXT-TMP: sulfamethoxazole –trimethoprim; AMP: ampicillin; CLI: clindamycin; GEN: gentamicin.

The results are shown in Figure 6. Within them, it is important to highlight that 100% of *E. coli* strains isolated from urinary tract infections (UTI) in cats were resistant to ampicillin and amoxicillin–clavulanic acid, whereas for dogs, the figures were below 20% [31].

#### 3.2.5. Norway (NORM-VET)

NORM-VET reports on the use of antimicrobial agents and occurrence of AMR in Norway. This programme was launched in 2000, coordinated by the Norwegian Veterinary Institute commissioned by the Norwegian Food Safety Authority [33].

In this report, AMU is expressed as kg of active substance sold for companion animals (cats and dogs) and corresponds to 360 kg. The most active sold substances were from the penicillin group, along with first-generation cephalosporins and trimethoprim, of which the most sold antimicrobial was the combination of amoxicillin and clavulanic acid [33].

**Figure 7:**
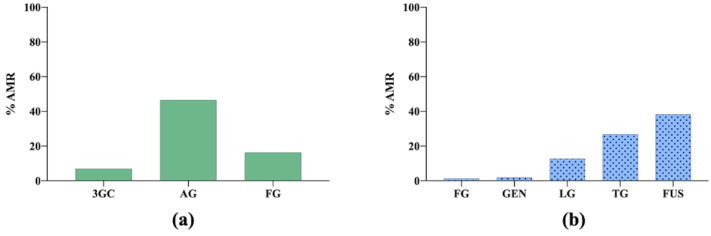
(**a**) Antimicrobial resistance (AMR) prevalence in *Escherichia coli* isolated from dogs in Norway. 3GC: third-generation cephalosporins; AG: aminopenicillins group; FG: fluoroquinolones group. (**b**) Antimicrobial resistance (AMR) prevalence in *Staphylococcus pseudintermedius* isolated from dogs in Norway. FG: fluoroquinolones group; GEN: gentamicin; LG: lincosamides group; TG: tetracyclines group; FUS: fusidic acid.

As seen in Figure 7, the highest AMR level in *E. coli* isolates was to aminopenicillins. However, for *S. pseudintermedius*, the highest AMR percentage was observed to fusidic acid [10,33].

#### 3.2.6. Sweden (SWEDRES-SVARM)

SWEDRES-SVARM reports on Swedish Antibiotic Sales and Resistance in Human Medicine (SWEDRES) and Swedish Veterinary Antibiotic Resistance Monitoring (SVARM). Data for this programme are provided by the Public Health Agency of Sweden and the National Veterinary Institute [34]. 

**Figure 8:**
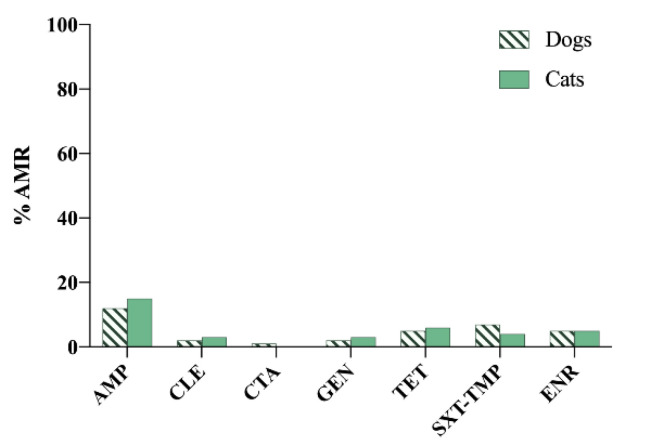
Antimicrobial resistance (AMR) prevalence in *Escherichia coli* isolated from dogs and cats in Sweden. AMP: ampicillin; CLE: cephalexin; CTA: cefotaxime; GEN: gentamicin; TET: tetracycline; SXT-TMP: sulfamethoxazole–trimethoprim; ENR: enrofloxacin.

**Figure 9:**
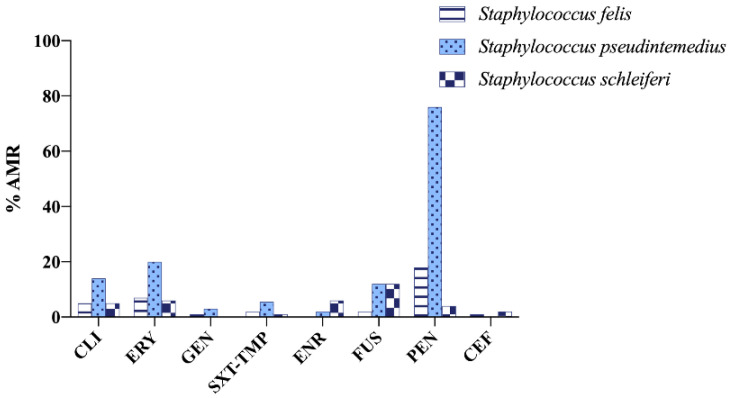
Antimicrobial resistance (AMR) prevalence in *Staphylococcus felis* isolated from cats and *Staphylococcus pseudintermedius* and *Staphylococcus schleiferi* isolated from dogs in Sweden. CLI: clindamycin; ERY: erythromycin; GEN: gentamicin; SXT-TMP: sulfamethoxazole–trimethoprim; ENR: enrofloxacin; FUS: fusidic acid; PEN: penicillin; CEF: cephalothin.

In Sweden, referring to companion animals, AMU data are only related to dogs [34]. In addition, sales only include orally administered medicines. The reduction in AMU has been remarkable, as sales of antimicrobials were 1200 kg in 2011, whereas, in the latest report, sales fell by more than 50%, with total sales of 589 kg in 2020 [34]. However, as in previous years, first-generation cephalosporins, aminopenicillins and lincosamides were the groups with the highest sales [34]. The following figures show the most relevant results reported (Figure 8 and Figure 9) [10,34]. 

#### 3.2.7. Switzerland (ANRESIS/ARCH-Vet)

ANRESIS/ARCH-Vet is the Swiss Centre for Antibiotic Resistance, which has drafted the Antibiotic Resistance Report in this country since 2019 [35]. 

Sales of antimicrobials in the last report were increased from the previous year to 775 kg of substances. Switzerland follows the trends in AMU of the rest of the European countries, with the penicillin group being the most consumed [35].

**Figure 10:**
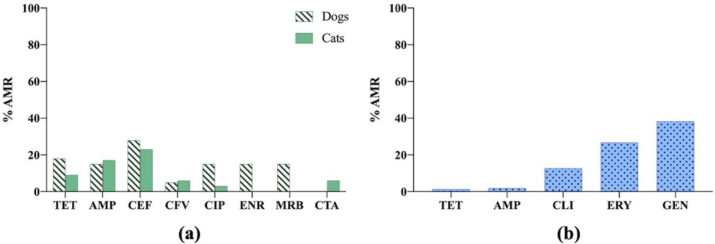
(**a**) Antimicrobial resistance (AMR) prevalence in *Escherichia coli* isolated from dogs and cats in Switzerland. TET: tetracycline; AMP: ampicillin; CEF: cephalothin; CFV: cefovecin; CIP: ciprofloxacin; ENR: enrofloxacin; MRB: marbofloxacin; CTA: cefotaxime. (**b**) Antimicrobial resistance (AMR) prevalence in *Staphylococcus pseudintermedius* isolated from dogs in Switzerland. TET: tetracycline; AMP: ampicillin; CLI: clindamycin; ERY: erythromycin; GEN: gentamicin.

*S. pseudintermedius* showed high AMR level to some antibiotics, such as gentamicin (~38%) and erythromycin (nearly 30%). However, in *E. coli*, higher AMR to cephalothin was observed in dogs and cats [10,35] (Figure 10). 

There are also AMR surveillance and monitoring programmes in the rest of the Member States, but they do not include companion animals. In this context, due to the need to harmonise all these data and the drive to set up a programme to control AMC and AMR together to guarantee that the data provided by all countries are equal and representative, the EU intends to launch the EARS-Vet to complement the current European Antimicrobial Resistance Surveillance Network coordinated by the ECDC. The combination of these programmes would provide the necessary “One Health” perspective.

### 3.3. Programmes in Development

One of the objectives of the GLG is to develop a coordinated system to control AMR and AMC surveillance and monitoring programmes [6]. Something similar is what the EU Joint Action on Antimicrobial Resistance and Healthcare-Associated Infections (EU-JAMRAI) aims to achieve with the EARS-Vet network in the EU Member States [5]. 

To get the programme up and running, a wide-ranging review was carried out to analyse the existing programmes in the EU. The information to establish this programme was collected from 13 EU countries, 10 of which have Monitoring and Surveillance programmes for AMR, AMC or both (the Czech Republic, Denmark, Estonia, Finland, France, Germany, Ireland, the Netherlands, Norway and Sweden), and the other 3 were in the process of setting up these programmes (Belgium, Greece and Spain). The shared information about the future scope of Belgium, Greece and Spain was defined through relevant national experts [5]. In this case, an important step was the definition of study areas, establishing which animal species/type of production/age categories/bacterial species/specimens/antimicrobials must be monitored.

Finally, EARS-Vet chose 6 animal species, i.e., cat, cattle, chicken (broiler and laying hen), dog, swine and turkey 11 bacterial species, i.e., *E. coli, Actinobacillus pleuropneumoniae, Klebsiella pneumoniae, Pasteurella multocida, Mannheimia haemolytica, S. aureus, S. pseudintermedius, Staphylococcus hyicus, Streptococcus suis, Streptococcus uberis* and *Streptococcus dysgalactiae* to be monitored [5]. Moreover, three panels of antibiotics were suggested, covering most of the combinations of importance in veterinary antimicrobial stewardship, following the EUCAST standards, recording minimum inhibitory concentration and reading antimicrobial susceptibility testing results using Epidemiological Cut-Off Values (ECOFFs) [5].

## 4. Alternatives to Antibiotic Use

As reported above, AMR represents one of the greatest threats to animal, human and environment health. In the past, antibiotics have been used not only to control infections, but also to prevent pathologies and improve the health status of the animal, without limits on their use or AMR prevalence control [36]. Therefore, high levels of AMR and multidrug-resistant bacteria are reported worldwide, so there is an ongoing quest to develop alternatives to antibiotic use in order to minimise the harm to public health [36]. This review describes the different strategies tested and approved to reduce infections and thus minimise the occurrence of AMR in companion animals.

### 4.1. Probiotics

Probiotics are live microorganisms that confer benefits to host health when administered in adequate doses [37]. There are also many microorganisms that have probiotic characteristics; the most common species to date are *Lactobacillus* spp., *Streptococcus* spp., *Lactococcus* spp. and *Bifidobacterium* spp. [37,38]. Probiotics should at least be capable of modulating the immune response or some physiological parameters of the host, treating or preventing infectious and inflammatory diseases and acting as biological preventive control agents [36,39].

One study evaluating the benefits of a probiotic, based on canine-derived *Bifidobacterium animalis*, in dogs with acute idiopathic diarrhoea showed that the use of the probiotic combined with the improvement of nutritional management reduced the need to administer metronidazole to the diseased dogs [37].

### 4.2. Prebiotics

Prebiotics are non-digestible compounds that are metabolised by gut microorganisms and modulate the composition and activity of the gut microbiota, providing benefits to the host’s physiological bacteria [40].

According to the results reported by De Souza et al. (2019), in dogs fed a mixture of fibre and prebiotics, no negative effect on nutrient digestibility and faecal quality was observed, and increased digestibility of food was reported. In addition, beneficial changes were observed in the faeces, which may indicate support of gut health. While the test substances caused slight changes in faecal microbial populations in adult healthy dogs, they had a significant effect on faecal metabolite physiology, demonstrating a possible microbial improvement in dogs fed diets supplemented with prebiotics. However, further research is needed to establish the optimal doses according to the age of the animals and the disease stages and to understand what conditions can be prevented or treated with prebiotic supplements [41].

### 4.3. Symbiotics

Symbiotics can strengthen the beneficial effects that probiotics and prebiotics have on their own, as probiotics use prebiotics as food sources to extend their survival in the digestive tract, increasing the digestibility and availability of certain nutrients such as vitamins, minerals, and proteins [42]. 

A study performed in dogs with acute diarrhoea, comparing the therapeutic effect of nutraceuticals and antibiotics on clinical activity, showed that the role of symbiotics in the positive effect seen in patients is unclear. Thus, more studies are needed both in vitro and in vivo, in companion animals, to further investigate the real effect of these supplements [43].

### 4.4. Postbiotics

Postbiotics are metabolites generated by the fermentation of probiotic bacteria in the gut [37]. In the last few years, they have had a great impact, as they have also been proposed as food supplements to regulate intestinal homeostasis instead of probiotics, since they reduce the possible risks of administering living bacteria [44]. Postbiotics are present in several species of *Bifidobacterium* (*B. breve, B. lactis, B. infantis*), *Bacteroides fragilis*, *E. coli* Nissle 1917 and *Faecalibacterium prausnitzii,* among others [45], and it has been reported that they improve the integrity of the mucosal gut barrier through different mechanisms, as well as modulate the secretion of inflammatory mediators [44,45].

In a study evaluating the postbiotic activities of *Lactobacilli*-derived factors in vitro, postbiotics were shown to have beneficial properties in relation to pathogen-induced inflammation and altered cytokine release [45].

However, as AMR is present in animal diseases and antibiotic treatment options sometimes do not work properly, efforts are being made to develop alternative treatments for ongoing infections [36]. 

### 4.5. Faecal Microbiota Transplantation

Faecal microbiota transplantation (FMT) involves the transfer of faeces from a healthy donor to the intestine of a diseased recipient with the aim of adjusting the gut microbiome of the diseased subject. The gut microbiota is seriously affected by the use of antibiotics or by inflammatory gastrointestinal diseases treated with antibiotics, which further aggravates a pathology [46]. 

Therefore, FMT is sometimes the only and last viable option [46,47]. Although more studies on FMT are needed, published clinical case reports for companion animals showed the improvement and restoration of the animal microbiota [48]. After treatment, animals recovered appetite and body weight, and the treatment might also help restore the integrity of the intestinal barrier, along with promoting the complete disappearance of the gastrointestinal and systemic symptomatology [46,47].

### 4.6. Bacteriophage Therapy

Bacteriophages (or “phages”) are viruses that possess the natural characteristic of specifically targeting and killing bacteria [49]. One of the advantages of phages is their ability to adapt to bacterial strains due to decades of co-evolution, which is why they are considered ‘adaptive drugs’ [49]. Moreover, their ability to lyse different bacteria strains has been reported. Although phages are considered a promising tool as an alternative to antibiotics, in veterinary medicine studies have been focused on food-producing animals, so there are only a few in vivo studies in companion animals [49,50].

Although most studies have been conducted in vitro, a clinical trial was carried out to study the treatment of otitis in dogs, caused by *Pseudomonas aeruginosa*, with a mixture of bacteriophages [40]. The outcome showed that bacteriophages improved the ear condition and all the dogs remained afebrile. In addition, the treatment scope for bacteriophages is limited, as the ear microbiota should be respected, so mixtures of bacteriophages are required to cover a wide range of bacteria [51].

All the information collected in this section is summarised in Table 1.

## 5. Antimicrobial Therapy and Its Impact on the Gut Microbiota

The biotic family explained above has played an essential role in complementing the prophylaxis and treatment of different diseases [38,52]. However, the impact of the antimicrobial therapy should be taken into account, as well as the influence of food choices on the microbiome and animal health in companion animals [39]. The typical formula for animal feeds is a balance between carbohydrates, proteins and fats and increasingly includes microbiome-targeted ingredients such as prebiotics or postbiotics. In animals, as in humans, food serves as a substrate for the bacterial microbiota, defining the composition and metabolites that will result from digestion [39]. Indeed, the gut microbiota could play an important role in the protection of the animal through *colonisation resistance* [53]. This term refers to the protection provided by the microbiota against the implantation of enteric pathogens and subsequent infections [53].

The gut microbiota lives in symbiosis with the host and is involved in its health status. The bacteria that make up the microbiota protect themselves from pathogens by competing for essential metabolites and nutrients and inducing intestinal immune responses, as studies show that there is increased immune system activity in the gut than in all other lymphoid tissues combined [54]. The gut environment is also modulated by the microbiota, which maintains low pH and oxygen levels. However, the gut microbiota also depends on the organism’s ability to respond to and regulate the inflammation of the intestinal wall [54]. Moreover, some medical conditions are known to influence its alteration, producing dysbiosis [38].

The microbiota of companion animals is dominated by the phyla *Fusobacteria, Bacteroidetes*, *Actinobacteria, Firmicutes* and *Proteobacteria,* which represent more than 99% of the gut microbiota of dogs and cats [39,40,54]. The remaining bacterial groups are represented by the phyla *Chloroflexi*, *Tenericutes*, *Spirochaetes, Cyanobacteria*, *Verrucomicrobia* and a few unclassified bacterial groups [40]. At the genus level, the most prevalent are *Helicobacter* spp. [54]. Therefore, due to the importance of the microbiota composition for the host, the alternative tools seek not only to combat pathogen infections, but also to improve the microbiota balance [39,54]. 

## 6. Conclusions

In conclusion, dogs and cats are considered an important potential source of AMR, posing a risk to public health. However, there are no harmonised control and monitoring programmes established in the EU, highlighting the need to create a joint plan to combat the evolution of AMR in companion animals, encompassing this concern under the One Health concept. For this reason, the EARS-Vet network is currently being developed, with the aim of establishing the bacterial species to be monitored, the complete antibiotic panels for these bacteria and the type of procedure to be followed to analyse collected samples. Moreover, alternatives to the use of antibiotics in veterinary clinic practice are being developed, with the aim of modulating the intestinal microbiota and prevent the onset of diseases (probiotics, prebiotics, symbiotics or postbiotics), also considering treatments such as faecal transplantation and bacteriophages.

## Figures and Tables

**Table 1:** Summary of alternatives to antibiotics in companion animals.

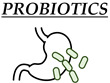	Living microorganisms that contribute to improve host health. - In a study in dogs with acute ideopathic diarrhoea, the use of probiotics with feed reduced the use of metronidazole compared to dogs treated without probiotics [28].
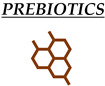	Non-digestible compounds used by beneficial gut flora. - Studies has shown that the use of prebiotics helps to improve food digestibility and gut microbiota [30].
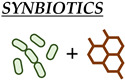	The synergistic combination of probiotics and prebiotics. - Although the use of synbiotics is promising, their full effects are not yet known and further studies are needed to assess their benefits in dogs and cats [28,32].
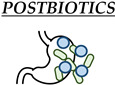	Compounds released by bacteria in metabolisation processes. - Some studies show that postbiotics help modulate the inflammatory response caused by a pathogen, however most studies are in vitro, so more in vivo studies are needed [33,34].
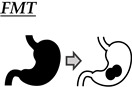	Transfer of faeces from a healthy patient to the intestine of a diseased patient to adjust the intestinal microbiota. - Studies have shown that animals regain body condition, appetite and help to restore the integrity of the intestinal wall after transplantation [35,36].
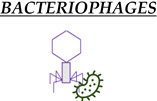	Viruses whose sole aim is to infect and attack bacteria. - Animals with infections treated with bacteriophages remained afebrile with a marked improvement of the affected area, however it was noted that given their small broad spectrum of action it is advisable to use combinations of bacteriophages [40].

FMT: Faecal Microbiota Transplantation.

## Data Availability

Not applicable.

## Abbreviations

3GC	Third-generation cephalosporins
AG	Aminopenicillins group
AMC	Amoxicillin–clavulanic acid
AMC	Antimicrobial consumption
AMP	Ampicillin
AMR	Antimicrobial resistance
AMU	Antimicrobial use
AMX	Amoxicillin
CEF	Cephalothin
CFV	Cefovecin
CHL	Chloramphenicol
CIP	Ciprofloxacin
CLE	Cephalexin
CLI	Clindamycin
CLSI	Clinical Laboratory Standards Institute
CPD	Cefpodoxime
CTA	Cefotaxime
CTF	Ceftiofur
DOX	Doxycycline
EARS-Vet	European Antimicrobial Resistance Surveillance Network in Veterinary Medicine
ECDC	European Centre for Disease Prevention and Control
ECOFFs	Epidemiological Cut-Off Values
EFSA	European Food Safety Authority
EMA	European Medicines Agency
ENR	Enrofloxacin
ERY	Erythromycin
ESVAC	European Surveillance of Veterinary Antimicrobial Consumption
EU	European Union
EUCAST	European Committee on Antimicrobial Susceptibility Testing
FG	Fluoroquinolones group
FMT	Faecal Microbiota Transplantation
FUS	Fusidic acid
GEN	Gentamicin
GLG	Global Leaders Group
GTI	Gastrointestinal tract infections
JIACRA	Joint Inter-Agency Analysis of Antimicrobial Consumption and Antimicrobial Resistance
LG	Lincosamides group
LM	Lincomycin
MAR	Marbofloxacin
MRSA	Methicillin-resistant *Staphylococcus aureus*
MRSP	Methicillin-resistant *Staphylococcus pseudintermedius*
OIE	World Organisation for Animal Health
OXA	Oxacillin
PEN	Penicillin
spp.	Species
SXT-TMP	Sulfamethoxazole–trimethoprim
TET	Tetracycline
TG	Tetracyclines group
UTI	Urinary tract infections
WHO	World Health Organisation
